# The Psychology Behind the Efficacy of a Buddy System for Enhancing Retention in Dermatological Clinical Trials

**DOI:** 10.7759/cureus.72986

**Published:** 2024-11-04

**Authors:** Kelly M Frasier, Grace Herrick, Hiral Patel, Sriya Kakarla, Lana Danial, Sri Vaishnavi Konagalla, Niralee Patel

**Affiliations:** 1 Dermatology, Northwell Health, New Hyde Park, USA; 2 Dermatology, Alabama College of Osteopathic Medicine, Dothan, USA; 3 Dermatology, Brody School of Medicine, East Carolina University, Greenville, USA; 4 Dermatology, University of Texas Health Houston McGovern Medical School, Houston, USA; 5 Dermatology, Andrew Taylor Still University School of Osteopathic Medicine, Mesa, USA; 6 Dermatology, Nova Southeastern University, Fort Lauderdale, USA; 7 Dermatology, University of Florida, Gainesville, USA

**Keywords:** academic dermatology, buddy system, clinical researcher, clinical trials in all phases (i-iv), psychosocial impact

## Abstract

Retention in dermatological clinical trials is critical for ensuring the validity and reliability of study outcomes, yet participant dropout remains a pervasive challenge. In this analysis, the primary aim is to explore the psychological mechanisms underpinning the efficacy of a buddy system in enhancing retention rates in such trials. The buddy system, where participants are paired with peers who have previously participated in similar clinical trials offers mutual support and leverages the principles of social bonding, accountability, and shared experience to foster commitment and reduce attrition. The novelty of this approach lies in its focus on the psychological and emotional dimensions of trial participation, rather than solely on logistical or incentive-based strategies. Implementing a buddy system draws on theories of social support and motivation, suggesting that the presence of a buddy can alleviate anxiety, increase perceived value, and enhance the overall clinical trial experience. Future research should investigate the long-term effects of buddy systems across diverse demographic groups and dermatological conditions, as well as potential adaptations for virtual trial settings. In conclusion, integrating a buddy system into dermatological clinical trials represents an innovative strategy that could significantly improve retention, thereby strengthening the robustness of clinical data and advancing dermatological research.

## Introduction and background

Participant retention plays a pivotal role in ensuring the validity and reliability of clinical trial data, which serves as the foundation for accurate research findings and robust statistical analysis. In dermatological clinical trials, participant dropout rates present a significant challenge, often jeopardizing the integrity of study outcomes. The nature of these trials - frequently involving intricate, lengthy, or uncomfortable procedures - can contribute to high attrition rates, underscoring the need for innovative retention strategies that address both logistical and psychological factors. Evidence suggests that retention improves when participants feel engaged and valued, such as through the involvement of senior investigators and proactive trial management [1]. These approaches help mitigate dropout and enhance the quality of the data collected, ensuring that the conclusions drawn are reliable and meaningful.

This paper explores the psychological mechanisms that underpin the efficacy of buddy systems in enhancing retention rates within dermatological clinical trials. While traditional retention strategies, such as financial incentives and accommodation, have shown moderate success, they often neglect the emotional and psychological dimensions of participant engagement. For example, health system navigator interventions, which emphasize social support and self-efficacy, have proven highly effective in other medical settings [2]. By incorporating social support and shared experiences, buddy systems offer a more holistic approach to participant retention. This model addresses not only logistical concerns but also the emotional and psychological needs of participants, which are often key contributors to dropout.

Buddy systems enhance retention by fostering accountability, reducing anxiety, and promoting strong social cohesion among participants. Accountability arises from the sense of responsibility participants feel towards their peers, motivating adherence to trial protocols and attendance at appointments. This peer support system not only minimizes the likelihood of dropout but also elevates the overall trial experience, making the participation more engaging and less isolating. The emotional reassurance provided by a peer who has undergone a similar experience can help alleviate the anxiety related to medical procedures and uncertainties. In dermatological trials, where participants may face additional psychological burdens due to the visible nature of skin conditions, having a buddy who understands and shares these challenges can be invaluable [3]. The shared journey through the clinical trial process transforms what could be a solitary and stressful experience into one of mutual support and collaboration, thereby increasing the likelihood of sustained engagement. 

The implementation of a buddy system requires thoughtful planning to ensure that participants are paired effectively based on factors such as demographic similarities, the nature of their dermatological conditions, and their personal experiences. This tailored approach ensures that the buddy relationship is meaningful, enhancing the support provided. Structured check-ins and dedicated communication channels can facilitate ongoing interaction, which is critical for the system’s success [4]. Furthermore, as dermatological clinical trials increasingly incorporate virtual elements, the buddy systems can be adapted to both in-person and decentralized, remote trial formats. This adaptability is essential, particularly in the evolving landscape of clinical research, where flexibility is key to maintaining participant engagement across diverse trial settings. By creating a trial environment where participants feel valued and supported, buddy systems can significantly enhance retention rates, contributing to more reliable and comprehensive study outcomes [5]. Ultimately, the effectiveness of these systems hinges on their capacity to address the various needs of participants, ensuring their continued involvement from the beginning to the end of the trial.

By integrating buddy systems into the framework of dermatological clinical trials, researchers can address many of the limitations associated with traditional retention strategies. This innovative approach offers a comprehensive solution that goes beyond addressing logistical barriers, focusing instead on the psychological needs of participants. As dermatological clinical trials continue to struggle with high dropout rates, exploring the utility of buddy systems could be a valuable tool for improving participant retention. The long-term success of these systems, however, will depend on their ability to adapt to the diverse needs of participants and the evolving formats of clinical trials. Continued research into the psychological impact of buddy systems, particularly in diverse demographic groups and dermatological conditions, will be critical to optimizing their implementation [1]. Moreover, as trials increasingly shift toward virtual settings, the flexibility of these systems will be paramount in ensuring their efficacy in various research environments. Ultimately, the use of buddy systems represents a promising avenue for enhancing participant retention and improving the overall quality of dermatological clinical research.

## Review

Retention challenges in dermatological clinical trials

Dropout rates in clinical trials vary considerably depending on the condition under study and the trial design [6]. For instance, a pooled analysis of 10 clinical trials on etanercept for psoriasis revealed that the perceived lack of efficacy drove a significant steep rise in dropout rates between days 75 and 85 [7]. Although data on dropout rates specific to dermatology trials is limited, understanding these dynamics is essential for designing studies that retain participants and yield reliable results. High dropout rates can undermine the trial's validity by introducing bias and reducing statistical power, particularly when participants who experience adverse events or perceived inefficacy are not adequately accounted for in the analysis [8,9]. Addressing these challenges requires careful consideration of patient-centered factors, such as treatment expectations and perceived benefits, to improve retention. 

Several factors contribute to participant attrition in dermatological trials. Inconvenience, particularly the frequency of in-person visits, poses a significant challenge [10]. Lack of motivation often arises from participants’ frustration over slow or limited improvements, especially in chronic skin conditions where visible progress is closely monitored. Discomfort with procedures, such as skin biopsies or frequent blood draws, further exacerbates dropout rates. For example, in atopic dermatitis trials, the application of study medications may result in temporary skin irritation, increasing the likelihood of dropout. Existing retention strategies - such as financial incentives, reminders, and accommodations - offer some support but are often insufficient for addressing deeper emotional and psychological concerns [11,12]. These strategies may improve participation in the short term but if they do not address the participants' underlying emotional needs, their long-term effectiveness remains limited.

Psychological mechanisms in the buddy system

Current strategies frequently overlook the psychological and emotional needs of participants, particularly in dermatology where visible skin conditions can result in significant psychological distress. Participants in dermatological trials often experience anxiety that practical support alone does not alleviate [13]. Furthermore, as the participants' understanding of the trial’s importance diminishes over time, ​​their motivation tends to wane, leading to higher dropout rates. The transactional nature of financial incentives and reminders may further erode the sense of partnership between researchers and participants, weakening long-term engagement [11]. Establishing peer connections within trials may help mitigate this decline in motivation by reinforcing the participants’ commitment to both the study and their peers.

A more patient-focused approach is needed to address these limitations and improve retention. Incorporating patient-reported outcomes, providing ongoing education about the trial’s impact, and fostering a sense of community among participants are essential strategies [11]. The buddy system leverages peer relationships to enhance emotional support and trial engagement by tapping into the fundamental human needs for social connection and shared experiences. These peer relationships create a supportive environment where participants feel validated, which is especially valuable in dermatological trials where psychological burdens can be profound. The effectiveness of social support in increasing resilience during stressful situations is well-documented [14]. In the context of clinical trials, a buddy system could help participants cope with the stresses and challenges associated with trial participation. By promoting social bonds, it can enhance resilience, improve retention, and contribute to more robust trial outcomes. Evidence from healthcare settings supports the effectiveness of peer support [15,16]. In dermatological trials, where psychological impacts are particularly pronounced, peer relationships can normalize the challenges of living with skin conditions and participating in a trial, fostering a sense of community and belonging.

Accountability is another crucial aspect of the buddy system. Participants who have a peer mentor often feel a greater sense of responsibility, motivating them to adhere to trial protocols. This effect has been observed in other medical settings. For instance, peer-to-peer (PTP) mentoring in dialysis patients has been shown to reduce missed treatments, with participants reporting that encouragement and shared experiences kept them focused and committed [17]. Similarly, in dermatological trials, peer support can reinforce treatment adherence and improve the commitment of the participants. This effect may even be seen in long-term trials involving multiple interventions. Social support can also sustain the engagement of participants who may have low expectations of treatment success. Peer relationships can inspire hope and resilience, as seen in kidney transplant patients who credit peer support for motivating them throughout the demanding interventions [18]. Integrating peer support within the clinical trial process offers continuous emotional encouragement, helping participants maintain their commitment to completing the study.

Additionally, the emotional reassurance provided by a buddy can alleviate anxiety and uncertainty associated with medical conditions and trial procedures. Chronic skin conditions often impose psychological burdens on the patients. This goes beyond the physical symptoms and includes self-esteem issues stemming from visible lesions [19]. Connecting trial participants with peers who have successfully navigated similar experiences can help them manage their emotional responses, enhance their self-esteem, and reduce stress [20]. Furthermore, peer support can empower participants by improving their knowledge about trial processes and reducing the feelings of uncertainty, ultimately boosting their engagement and perseverance. This combination of emotional support and practical knowledge reinforces the bond between participants, creating a sense of shared purpose and accountability. The buddy system transforms the clinical trial journey from an individual experience into a collective one. When participants share personal challenges and exchange information, they create a collaborative and supportive community focused on mutual well-being [21]. This sense of camaraderie fosters solidarity, reduces anxiety, and empowers participants. In dermatological trials, emotional support from a peer can help participants feel less isolated and more validated, ultimately increasing their motivation to remain engaged in the trial.

Implementation of the buddy system

The design of an effective buddy system for clinical trials requires thoughtful pairing strategies to maximize the benefits of peer support. Participants should be matched based on various criteria to enhance compatibility and relevance in their interactions [22]. One approach is to consider demographic factors such as age and gender, ensuring participants feel more comfortable with their buddy. Additionally, pairing individuals with similar dermatological conditions or those participating in the same type of trial ensures that they share relevant experiences and challenges. Matching based on the severity of the condition can prove beneficial, as it allows for a more tailored exchange of strategies and coping mechanisms that align with the intensity of symptoms each participant is experiencing. By considering these factors, the buddy system can improve the quality of support and elevate the overall experience for participants in the dermatological clinical trials.

Integrating buddies into the clinical trial process requires careful planning to establish structured check-ins and accessible communication channels, which facilitate regular interactions between participants and their assigned buddies. These touchpoints might include weekly video calls, text messaging, or online updates where buddies can discuss progress, share experiences, and address any concerns [23]. Using dedicated platforms or secure messaging apps can further streamline communication, ensuring participants can easily connect and support each other. It is essential that these communication tools are both user-friendly and accessible to maintain engagement and foster a supportive environment. By thoughtfully incorporating these logistical elements, the buddy system enhances the trial experience and contributes to improved participant adherence.

Pairing new participants with those who have completed similar clinical trials can be especially important in addressing the psychological challenges associated with clinical trials. Dermatology, in particular, can be daunting due to the visible nature of skin conditions and their treatments, amplifying feelings of self-consciousness and stress [24]. New participants may feel overwhelmed or anxious by how their condition and treatment could affect their appearance and daily life. By connecting them with mentors who have successfully navigated similar trials, participants gain empathetic support and practical advice from someone who has faced and overcome similar challenges. This personal connection can significantly reduce feelings of isolation and anxiety, providing reassurance that their concerns are shared and understood. Mentors who have managed side effects or dealt with the emotional impact of visible skin changes can offer crucial insights and encouragement [25]. This support not only helps participants feel more secure and prepared but also fosters camaraderie and emotional solidarity. Addressing these psychological aspects through peer mentorship can enhance overall well-being, boost confidence, and cultivate a more positive outlook towards the trial experience.

Peer mentorship within clinical trials has the potential to significantly enhance the participants' sense of security and their understanding of the trial process. Participants benefit from both practical insights and emotional support, which can help alleviate anxiety and boost confidence. Mentors help demystify the trial experience by sharing firsthand knowledge about managing treatment regimens, coping with side effects, and adhering to protocol requirements [23]. This guidance fosters a clearer understanding of what to expect, leading to increased adherence and overall satisfaction. Research shows that mentorship and peer support can positively impact participant engagement, reduce stress and improve outcomes [26]. The effectiveness of peer mentorship has been demonstrated in studies involving patients with diabetes mellitus, HIV, and kidney disease, underscoring its potential in clinical trials [27]. By enhancing the participants' understanding of the trial process and providing emotional support, peer mentorship plays a crucial role in improving the clinical trial experience and its overall outcomes. 

Incorporating technology

Incorporating virtual buddy systems into dermatology trials shows promising potential. With the rise of digital and decentralized clinical trials, these systems can leverage advanced mobile applications to provide valuable support to patients. Research highlights that positive outcomes are dependent on the buddies being actively engaged, underscoring the importance of pairing participants who are committed to the peer mentor experience throughout the trial [28]. Virtual buddies can assist participants by helping them navigate complex trial protocols, providing reminders for medication adherence, and sharing personal experiences to ease the challenges of the trial process. This approach not only enhances participant retention but also fosters a sense of community and shared purpose, reducing the isolation that many participants may feel.

Online platforms designed for communication and emotional support also play a significant role in virtual settings. Such virtual interactions are especially important in remote trials or for patients managing chronic conditions [24]. These patients can feel disconnected from the broader healthcare community and from others facing similar challenges. Virtual buddy systems and online platforms bridge this gap by providing consistent, real-time communication and emotional support, as demonstrated in dermatological research [23,29]. Depression and anxiety are common concerns for patients with dermatological conditions, and online support communities have been shown to reduce depressive symptoms and improve quality of life [30]. This mutual exchange of support fosters solidarity, offering patients comfort in knowing they are not alone in their struggles. Moreover, they can benefit from the shared knowledge and encouragement from their peers, which can reduce feelings of loneliness, improve emotional resilience, and contribute to a more positive overall experience. 

The role of social support in enhancing clinical trial outcomes

Social support mechanisms, including peer assistance and mentorship, are increasingly being recognized for their potential to improve participant involvement and retention in clinical trials. This section of the review examines the effectiveness of these systems across various medical fields and evaluates their impact compared to other areas where similar approaches have shown positive results. In oncology, peer support has demonstrated significant benefits. Kiemen et al. explored the outcomes of PTP support for cancer patients [31]. Their findings indicated that peer support programs, which include emotional, informational, and psychosocial support from other cancer survivors, have several benefits. Specifically, these programs helped reduce anxiety, improved coping mechanisms, and fostered a sense of hope among participants. Although the review found only modest improvements in depression and anxiety, PTP support has been linked to better cancer-specific quality of life outcomes, particularly in face-to-face settings [31]. This aligns with the general understanding that peer support can improve adherence to treatment and enhance patient satisfaction by offering emotional and psychological support.

Similarly, peer support has been shown to be highly effective in diabetes management. Powers et al. conducted a review of multiple studies and concluded that peer support significantly improved diabetes self-management and glycemic control [32]. Individuals who participated in peer support networks were more likely to follow self-care recommendations and achieve better blood sugar control [32]. The positive impact of peer involvement is a clear indicator of its potential for broader application in chronic disease management, and suggests that such support systems can improve both health outcomes and patient engagement.

Peer support models, particularly buddy systems, have shown promise in enhancing adherence to long-term treatment regimens, especially in chronic illnesses like HIV. In a global network meta-analysis by Kanters et al., the effectiveness of peer-based interventions on antiretroviral therapy (ART) adherence was rigorously evaluated. The study included 22 randomized clinical trials and found that peer support, when combined with additional interventions like telephone follow-ups, significantly improved ART adherence [33]. For instance, peer supporter interventions paired with telephone support showed a substantial improvement in adherence rates, with an odds ratio of 4.87 compared to standard care. However, peer support alone yielded only modest improvements, and the analysis did not find significant effects on viral suppression, possibly due to a limited number of trials assessing that outcome. Despite this, the study’s findings underscore the value of peer support in reinforcing treatment adherence, suggesting that such models could be effectively applied to other chronic conditions, including dermatology [33]. Given that healthcare systems already implement many such interventions, such as counseling and digital reminders, formalizing buddy systems could help bridge existing gaps, maximizing both emotional support and treatment adherence in chronic disease management.

Similar success has been observed in other areas, such as education and professional settings. Topping et al. reviewed the effectiveness of peer tutoring in educational contexts and found that such methods significantly enhance student performance and retention [34]. Peer tutoring not only improved academic outcomes but also created a supportive learning environment, lowering dropout rates [34]. These results align with the benefits of peer support observed in clinical research. In professional settings, mentorship programs have been associated with greater job satisfaction and lower employee turnover. Allen et al. found that employees with mentors reported higher levels of satisfaction and were more likely to remain with their companies, illustrating how the principles of mentorship can enhance retention in various fields [35]. Adopting a peer support approach may hold promise for addressing the specific retention challenges faced in dermatological clinical trials.

Khan et al. found that adolescents participating in team sports experienced greater mental health benefits, such as reduced depressive symptoms and improved life satisfaction, compared to those who did not engage in sports or participated alone [36]. The support and shared experiences in team sports mirror the benefits of peer systems in clinical settings, where collaboration fosters accountability and encouragement [36]. These findings highlight the broad applicability of social support systems, showing that whether in sports or in clinical trials, such peer interactions enhance motivation, resilience, and overall well-being. The consistency of these results across different contexts demonstrates the universal value of social support mechanisms​.

In dermatology, while social support systems remain less documented, the research by Strober et al. sheds light on the critical importance of aligning patient-reported outcomes (PROs) with clinical treatment goals, particularly for chronic skin conditions like psoriasis [37]. Their research emphasized that achieving comprehensive skin clearance is vital not just for addressing the physical symptoms of psoriasis but also for improving overall patient quality of life. The study also highlighted that even small residual patches of the disease can have a disproportionate impact on the patient's mental health and day-to-day functioning, suggesting that incomplete clearance can lead to reduced satisfaction and engagement with the treatment process [37]. The research pointed to a broader need for dermatologists to place more emphasis on patient perspectives when setting treatment goals. PROs, which capture the subjective experience of patients, should be prioritized alongside traditional clinical measures like the Psoriasis Area and Severity Index (PASI). This approach acknowledges that the patient’s experience of symptoms, especially those affecting psychosocial aspects such as anxiety and social interaction, is crucial to successful treatment outcomes. Strober et al. argued that patient satisfaction and adherence to treatments are strongly tied to how well these subjective symptoms are addressed [37]. Moreover, as dermatologists begin to explore social support systems, such as support groups or peer mentorship, they may find parallels to other areas of medicine where these systems have enhanced patient adherence and quality of life. Strober et al. noted that such support could potentially bridge the gap between clinical expectations and the patient's lived experience of their condition [37]. As dermatological care continues to evolve, integrating both clinical and psychosocial support systems could be a pivotal strategy in improving patient retention and satisfaction across a range of skin conditions​

Research across various domains underscores the significant benefits of incorporating social support systems to enhance participant involvement and retention. Although the application of these systems in dermatology is still in its early stages, current evidence suggests that integrating support mechanisms could improve patient adherence and satisfaction. The success of peer support in managing chronic diseases, improving educational outcomes, and enhancing job satisfaction provides a strong foundation for further exploration in dermatological trials. Social support systems have the potential to address both the emotional and practical needs of patients. As dermatology research continues to evolve, adopting these strategies could lead to more effective patient care and better clinical outcomes. Further exploration and adoption of these support mechanisms are likely to yield important insights into improving patient experiences in dermatological clinical trials.

Areas for future consideration

The buddy system, which pairs participants with peers for mutual support, holds promise for improving retention rates in clinical trials. However, longitudinal studies are needed to assess its long-term impact on retention. Such studies would provide valuable insights into how buddy systems influence participant engagement over extended periods, particularly in trials that require long-term commitment. Currently, there is limited information on the specific impact of buddy systems on retention in clinical trials, with most existing studies focusing on individual strategies like financial incentives or logistical support rather than peer support mechanisms. This gap highlights the potential value of buddy systems, especially in trials involving chronic conditions or lengthy follow-up periods, where traditional retention strategies may fall short. Longitudinal research could determine whether buddy systems effectively reduce dropout rates by fostering community and shared experience among participants. These studies could also examine how such systems influence participants' perceptions of the trial, satisfaction levels, and long-term engagement. Understanding these aspects is crucial, as participant satisfaction and sustained involvement are key factors in the success of clinical trials [38]. The buddy system may enhance these factors by providing emotional support and fostering a sense of belonging, but empirical evidence on its effectiveness remains limited. This underscores the need for further investigation into the benefits of buddy systems in various clinical contexts. 

While the buddy system has the potential to enhance retention and engagement, its impact may vary across different demographic groups, requiring more targeted research. There is a pressing need to examine how buddy systems affect diverse populations to ensure inclusivity in clinical trials. Without careful consideration, peer-pairing strategies could inadvertently exclude or disadvantage certain demographic groups, undermining the inclusivity and effectiveness of the intervention. This potential for bias reinforces the importance of designing buddy systems that accommodate a wide range of participants. Additionally, ensuring inclusivity in trial design is essential, particularly when implementing buddy systems. Researchers must address potential barriers to participation for underrepresented groups by recruiting a diverse pool of buddies and providing them with the necessary training to mentor effectively [39,40]. Rather than focusing solely on demographic similarities, buddy-pairing criteria should emphasize shared experiences, common challenges, and complementary strengths that can foster meaningful connections. This approach promotes equity and helps ensure that all participants benefit from the support system, regardless of their background.

Adapting buddy systems for virtual trials in dermatology presents both challenges and opportunities as the field increasingly shifts toward digital research methodologies. One concern is the potential impact that buddy systems could have on trial outcomes, as introducing peer support may inadvertently influence participant behavior or responses. This could raise questions about the integrity of the data, as buddy interactions might affect variables that are critical to the study. However, buddy systems can be strategically implemented to play a supportive role without directly interfering with the core elements being measured. By focusing on enhancing participant retention and engagement rather than influencing clinical outcomes, buddy systems could serve as an external reinforcement to keep participants motivated throughout the trial.

To achieve this balance, the design and implementation of buddy systems must be carefully thought out. Clear boundaries between the buddy system's support functions and the intervention being studied should be established. For instance, buddy interactions can be confined to providing emotional support, answering logistical questions, or sharing personal experiences, ensuring that these elements do not interfere with the study's primary measurements. Additionally, rigorous trial designs can incorporate mechanisms to monitor and document buddy system interactions, ensuring transparency and mitigating potential biases. This might involve regular check-ins with participants or using standardized communication tools to limit unintentional influences on behavior that could skew trial results. Ultimately, the success of integrating buddy systems into virtual trials depends on striking the right balance between maintaining participant support and preserving the scientific rigor of the study. With proper implementation and monitoring, buddy systems can boost retention rates in virtual dermatology trials without compromising the quality or validity of the research outcomes.

Virtual clinical trials (VCTs) are particularly well-suited for dermatology, given the visual nature of skin conditions and the convenience of home-based participation. By reducing logistical burdens, VCTs have the potential to decrease dropout rates and improve overall trial efficacy [41]. To effectively integrate buddy systems into virtual dermatology trials, researchers must find ways to preserve the benefits of peer support in a digital environment. One approach is to create virtual peer groups or pairs, facilitated through secure online platforms, offering the same emotional support and shared experiences as traditional systems. Various technological tools can enhance communication in these virtual environments. Secure video conferencing platforms can replicate face-to-face interactions, fostering connections, while mobile applications can serve as centralized hubs for communication, sharing experiences, and accessing trial-related information. By leveraging these technologies, virtual buddy systems have the potential to maintain participant engagement and improve retention rates in VCTs.

However, challenges in adapting buddy systems for virtual trials must be addressed. These include ensuring all participants have equal access to the necessary technology, protecting sensitive health information shared between buddies, and fostering authentic relationships in a virtual setting. To overcome these challenges, researchers could offer technology training for participants, ensure user-friendly interfaces, and incorporate both synchronous and asynchronous communication options to accommodate different preferences and schedules. As dermatology research continues to embrace VCTs, adapting buddy systems for digital environments offers a promising way to enhance participant support and retention. Future research should focus on developing and evaluating virtual buddy system interventions to determine their effectiveness in improving trial outcomes and participant experiences in dermatological VCTs.

## Conclusions

The implementation of buddy systems into dermatological clinical trials offers a compelling and multifaceted solution to the persistent issue of participant retention by addressing both psychological and emotional needs that traditional logistical strategies often fail to meet. The buddy system enhances participant engagement by fostering meaningful social bonds, which provide crucial emotional support and a sense of belonging, reducing the isolation often felt during trial participation. It also heightens accountability, as participants are more likely to adhere to trial protocols and remain committed when they have a peer to whom they are accountable. Additionally, the presence of a buddy can significantly alleviate the anxiety associated with medical procedures, trial uncertainties, and the overall commitment required, thereby mitigating the stress that can lead to early withdrawal. For these benefits to be fully realized, it is essential to integrate buddy systems systematically into clinical trial protocols, with careful consideration given to the pairing of participants based on shared experiences and demographics as well as to the establishment of effective communication and support mechanisms. Supporting ongoing research to evaluate the long-term effects of buddy systems and refine their implementation is also critical. This includes exploring their impact across diverse demographic groups and adapting them for virtual or decentralized trial formats. By embracing and optimizing buddy systems, dermatological research can achieve higher retention rates, and more reliable data, ultimately advancing the field by producing more robust and meaningful research outcomes.
